# A Prototype System for Measuring Microwave Frequency Reflections from the Breast

**DOI:** 10.1155/2012/851234

**Published:** 2012-04-24

**Authors:** J. Bourqui, J. M. Sill, E. C. Fear

**Affiliations:** Department of Electrical Engineering, Schulich School of Engineering, University of Calgary, Calgary, AB, Canada T2N 1N4

## Abstract

Microwave imaging of the breast is of interest for monitoring breast health, and approaches to active microwave imaging include tomography and radar-based methods. While the literature contains a growing body of work related to microwave breast imaging, there are only a few prototype systems that have been used to collect data from humans. In this paper, a prototype system for monostatic radar-based imaging that has been used in an initial study measuring reflections from volunteers is discussed. The performance of the system is explored by examining the mechanical positioning of sensor, as well as microwave measurement sensitivity. To gain insight into the measurement of reflected signals, simulations and measurements of a simple phantom are compared and discussed in relation to system sensitivity. Finally, a successful scan of a volunteer is described.

## 1. Introduction

Microwave imaging has been proposed as an alternative breast imaging modality [1]. The basic premise is that different tissues in the breast have different electromagnetic properties, and these differences may be exploited to create images. General approaches to active microwave imaging include microwave tomography [2] and radar-based methods [3–5]. Microwave tomography involves measuring signals transmitted through the breast and reconstructing images by matching measured data with signals obtained from simulated models containing iteratively updated property estimates. Microwave tomography has been tested with simulations and experimental measurements of phantoms (e.g., [6]) and simulations of realistic breast models [7]. Moreover, a research group at Dartmouth College has performed extensive patient studies with prototype systems. The resulting images have demonstrated average microwave frequency properties that increase with breast density [8], as well as agreement between features detected on microwave images and known clinical histories [9]. Radar-based microwave techniques create images by processing reflections of wideband or ultrawideband (UWB) signals from the breast. These images indicate the presence and location of significantly scattering objects. Testing of radar-based approaches has involved simulations with realistic breast models [3, 10], testing with phantoms [5, 11, 12] and early-stage clinical investigations [13]. To date, a group at Bristol University has reported imaging of patients using a multistatic radar system. Therefore, in spite of the growing body of literature related to microwave breast imaging, there are very few reports of work with patients or volunteers. This likely reflects the significant technical challenges involved in sensor design and implementation, measurement hardware, and development of patient interfaces.

In this paper, we describe a prototype system that is based on a monostatic radar approach and has been termed the TSAR (tissue sensing adaptive radar) method. The TSAR prototype system differs from previously reported prototype systems for microwave imaging in that a single antenna is scanned around the breast in order to collect data. A multistatic system inherently collects more information than its monostatic counterpart. On the other hand, a single-sensor method can be designed to produce a focused beam increasing the reflected power from small features. Given the potential high attenuation in breast tissues, this is likely beneficial for sensing smaller malignant regions. In addition, a monostatic system allows more relaxed requirements for the UWB sensor. A larger sensor permits using lower frequencies without limitations due to mutual coupling. The ability to place the sensor at an infinite number of locations around the breast is also very attractive in terms of adaptability to patients, as well as for image reconstruction performance. However these advantages are at the cost of a more complex positioning system and longer repositioning time compared to electronically switched antennas as in [13]. In order to assess the performance of our prototype system, a study is performed of the mechanical sensor positioning, as well as of the microwave measurement sensitivity and perturbation. This provides insight into the capabilities and limitations of the system. Next, we compare simulations and measurements of a simple phantom. While both simulations and experimental work have previously been carried out for tomography and radar-based imaging, only a few papers directly compare simulations and measurements of phantoms (e.g., [6, 14]). Our phantom represents the shape of the breast in a simplified way and consists of one material with an inclusion of a different material. Although the properties of the model differ from those of breast tissues, the phantom has stability in properties and shape that permit evaluation of the repeatability of results. In addition, the reflections from the phantom are interpreted relative to the system sensitivity. After validation, the prototype system is used to collect reflections from volunteers. To gain insight into these measurements, comparison with simulations of volunteer-specific breast models is attempted.

## 2. Prototype System and Procedure

### 2.1. System Description

**Figure 1:**
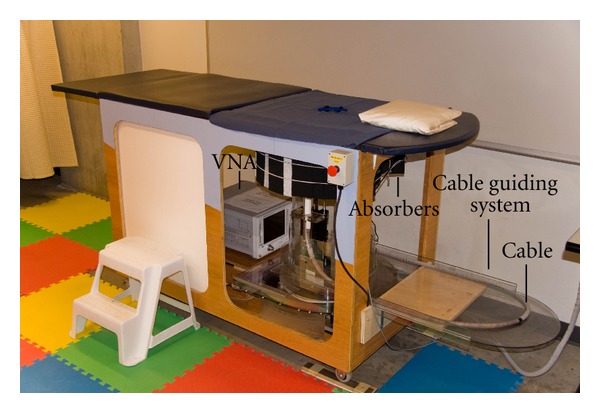
TSAR system used to scan volunteers.

The TSAR prototype system is shown in Figure 1. The prototype consists of a padded bed placed over a cylindrical tank filled with canola oil. The woman to be scanned lies prone on the bed, and a hole in the top of the bed permits one breast to extend into the tank.

**Figure 2:**
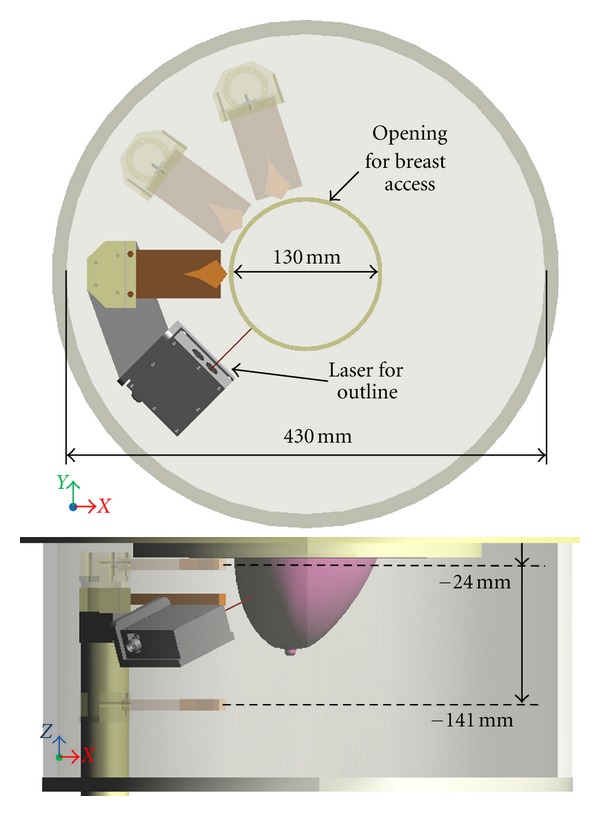
Top and side views of the TSAR prototype system tank with dimensions. Additional antenna locations are shown (shaded antenna body) to illustrate the tank rotation and arm movement. For better clarity, the laser is not shown for the additional antenna positions.

The cylindrical tank is filled with canola oil to improve the matching between the breast skin and the sensor attached to a positioning arm. The canola oil exhibits a relative permittivity of 2.5 with a conductivity below 0.04 S/m up to 12 GHz. A laser is also mounted to the positioning arm to record the breast outline. To scan the sensor around the breast, the arm moves vertically and the entire tank rotates. Dimensions of the tank and hole as well as antenna location are provided in Figure 2. The scanning region in the vertical (*z*) direction spans from 24 mm to 141 mm below the top of the lid. The circular opening in the lid has a diameter of 130 mm while the tip of the sensor is located 70 mm away from the center of the opening to avoid contact with the breast skin. To monitor the scan procedure, a camera is mounted on the side of the tank and transmits images to the operator.

Microwave measurements are collected with a custom antenna. The antenna utilized in this work is a balanced antipodal Vivaldi antenna with a director (BAVA-D) [15]. This antenna has a bandwidth (*S*_11_ better than −10 dB) from 2.4 to 18 GHz. The director narrows the beam of the antenna compared to a standard BAVA design, thus focusing more energy into the breast. Measurements are acquired with a vector network analyzer (VNA) (8722ES, Agilent Technologies, Palo Alto, CA, USA). The antenna is connected to the VNA via a 3 m long cable, and a guiding system helps to move the cable in a reproducible way. The cable guiding system is indicated in Figure 1. The system is calibrated at the end of the cable where the antenna is connected. Measurements are taken at 1601 points over the frequency range from 50 MHz to 15 GHz with a port power of −5 dBm. As discussed in Section 3, an intermediate frequency (IF) bandwidth of 1 kHz and averaging over 3 frequency sweeps are used to reduce the system noise floor. The resulting data are transformed into the time domain after weighting with the spectrum of the differentiated Gaussian pulse given by:

(1) $$V\left(t\right)=V_{0}^{*}{\left(t-t_{0}\right)}^{*}e^{-(t-t_{0})/\tau^{2}},$$

where *V*_0_ is used to adjust the amplitude of the pulse, *τ* = 62.5 ps, and *t*_0_ = 4*τ*.

### 2.2. Volunteer Scan Procedures

**Figure 3:**
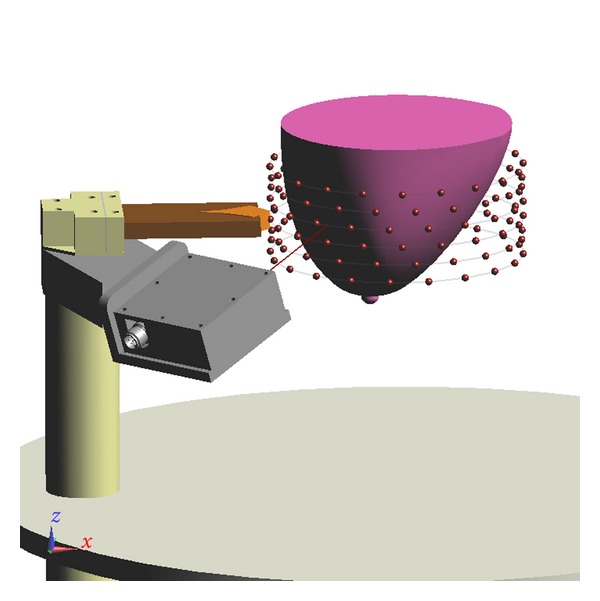
View of the scan pattern used for measurement. Each sphere corresponds to an antenna location. Antennas located on a common row are connected by lines.

We have scanned several volunteers with the prototype system (Study No. 21859, as approved by the University of Calgary Conjoint Health Research Ethics Board). Our study involves a TSAR scan of one breast, as well as a scan of both breasts with magnetic resonance (MR) imaging. During a TSAR scan, the antenna is physically moved to a number of locations encircling the breast at various elevations (Figure 3). Data collected at the same elevation are termed a row. For a complete scan, data are collected at a number of rows. For the volunteer scan, the number of rows, separation between rows, and number of antenna locations in a row are initially estimated with the MR images, then updated after observing digital images of the breast in the TSAR scanner. Our experience indicates that adjustments to TSAR scan patterns designed with MR images are necessary to compensate for the changes in breast shape and extent due to the flotation of the breast in oil. We note that the rotation of the tank and the vertical movement of the arm used to scan the antenna around the breast are both automated and actuated by step motors, which are controlled by a custom software code. The process of moving the sensors and collecting measurements takes less than 30 minutes for 1 breast scanned at up to 200 antenna locations.

The reflections are calibrated by performing two sets of measurements and then using responses from known objects to orient reflections in time. First, a scan is collected with the volunteer positioned in the scanner and another scan is acquired with an empty tank. To initially calibrate the data, the signals recorded with the empty tank are subtracted from signals recorded with the volunteer present. Identical antenna locations are used with both scans. Next, reflections from metal plates placed at two known distances from the antenna are collected. The differences in time of arrival of the two reflections are used to confirm the dielectric constant of the immersion medium. The known locations of the plates are also used to identify the reflection from the antenna aperture in the signals. The aperture reflection is then located in time in order to identify distances of objects relative to the end of the antenna.

Finally, the reflected signals are used to create images. First, the dominant reflections between the immersion liquid and object (e.g., oil/skin interface) are removed by approximating the reflections at a target antenna. For simple models such as the hemisphere used later in this paper, it is sufficient to use straightforward methods for this approximation. In this case, the reflections recorded at antennas located in the same row are time-shifted and scaled to match the target signal [16]. More sophisticated algorithms are typically required to deal with more complex scenarios. Next, 3D images are formed by scanning the focal point through the imaging region and using a time-shift-and-sum beamformer to identify components of the reflections at appropriate antennas that originate from the same physical location [16]. An estimate of the surface of the phantom is incorporated into this focusing procedure [17].

## 3. System Performance and Validation

As evident from the description in Section 2, the TSAR measurement system is rather complex. Many aspects of the system can alter the measurement quality, which in turn will influence the quality of the reconstructed images. We consider 3 different types of effects: (1) the positioning performance, (2) the microwave measurement sensitivity, and (3) perturbation. In this section, these different aspects are assessed or validated in order to define the overall system performance.

### 3.1. Positioning Performance

Correct positioning of the sensor is critical in two aspects. First, good mechanical precision is required for repeatability of measurements. As described in Section 2, each scan is calibrated with reference measurements collected during a scan with the exact same pattern but without the volunteer or patient present (empty tank). This operation removes the unwanted effects of the environment (e.g., reflections from the tank) from the measured signals. Therefore, good positioning repeatability is needed to guarantee that the unwanted effects are reproduced between the two scans. Second, good mechanical accuracy is necessary for proper image reconstruction as the signals are spatially focused based on the antenna positions. Good agreement between the desired and actual antenna positions in the scan is therefore required. The positioning precision and accuracy are related to the mechanical play and the ability to achieve the correct displacement; both of these parameters will be evaluated.

Two independent axes are used to bring the sensor into position, namely, the azimuth (°) (tank rotation) and the elevation (mm) (arm movement). Specifications of ±0.1° and ±0.1 mm for the displacement tolerance with a mechanical play of maximum 0.1° and 0.1 mm have been defined for each axis. These correspond to no more than 0.6 mm of error when identifying focal points in the worst-case scenario.

In order to validate these requirements, specific movement sequences are realized and expected positions are compared with a measurement of the actual position. For the elevation axis, the position is measured with a digital caliper attached to the moving arm. The assessment of the azimuth position is achieved by measuring the displacement on the outer edge of the rotating tank. Given the very large external diameter of the tank (520 mm), small angular displacements translate into large displacements at its outer edge. Note that the external diameter also includes a lip placed around the tank to collect excess oil, which contributes to the large difference when compared to the inside diameter given in Figure 2. This technique allows us to determine whether the azimuth movement passed or failed the specification, however no numerical values are extracted.

For the elevation axis, the validation shows that the displacement error is within tolerance with a maximum of ±0.07 mm and an average of ±0.04 mm. On the other hand, the mechanical play of the elevation axis is, in general, very close to the maximum allowed value and exceeded the limit in one of the test iterations. Therefore, an automated compensation of the mechanical play is implemented in the software used to control the TSAR prototype, showing significant improvement. The measured mechanical play results with and without software compensation are shown in Table 1.

All the azimuth tests passed the specification requirements successfully. However, movement with resolution of 0.25° creates a consistent displacement error that accumulates and creates larger positioning error. This behavior naturally occurs due to the intrinsic angular resolution of the step motor. This behavior is avoided by allowing displacements with a minimum resolution of 0.5°. 

### 3.2. Microwave Measurement Sensitivity

Since the reflections from internal breast tissues are expected to be very weak, good measurement sensitivity is a key aspect of the system. As described in Section 2, the calibrated data result from a subtraction of two successive scans: one with the volunteer present and one with an empty tank. Therefore, the sensitivity can be defined as the smallest signal that can be recovered after the subtraction operation. To assess the sensitivity of the microwave measurement system, a broadband load standard (Agilent 85052D) is connected instead of the antenna and two measured reflected signals are subtracted. Smaller differences correspond to better sensitivity. 

**Figure 4:**
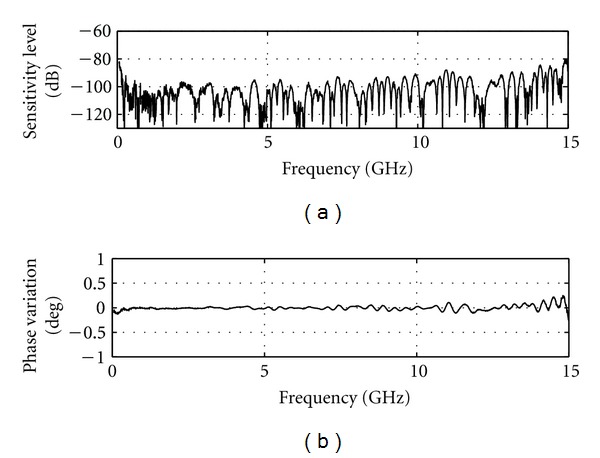
Sensitivity calculated using two successive static measurements.

The sensitivity is directly influenced by the measurement noise floor of the VNA receiver. Reduction of the IF bandwidth and averaging a number of measurements can significantly improve the noise level. The smallest IF bandwidth with a large amount of averaging would be ideal for sensitivity. However, these actions considerably increase the measurement time to impractical values. The maximum scan time for TSAR is set to 30 minutes for 200 measurements. Accounting for mechanical displacement time, the microwave measurement for each location has to be achieved in 8 seconds for a total of 26.6 minutes dedicated to the RF measurement. An IF bandwidth of 1000 Hz with averaging of 3 signals shows the best sensitivity among the combinations that fit the time criteria.  Figure 4 shows the sensitivity that is achieved with these settings and the broadband load attached. A sensitivity below −90 dB is achieved over almost the entire frequency band. The phase variation is below 0.2° with exception of the upper limit of the frequency band. This result can be considered as the best sensitivity that the system can achieve as the two measurements considered are collected in an ideal scenario in which no time elapsed and nothing moved between measurements. 

**Figure 5:**
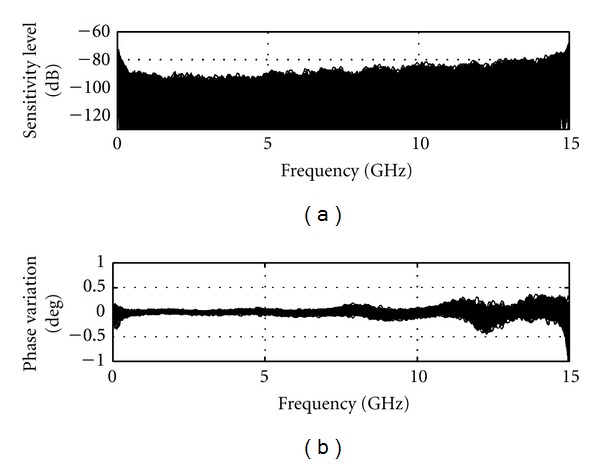
Sensitivity calculated based on a pair of 200 static measurements (no change of position) with a broadband load instead of the antenna. Measurement pairs are separated in time by 30 minutes to reproduce the time frame of two consecutive full TSAR scans.

The stability of the reflection measurement with respect to time will also influence the sensitivity. A 30-minute span occurs between the signals measured during the volunteer scan and the calibration scan. To evaluate the effect of this time delay on the sensitivity, 200 successive measurements of the broadband load are collected for two consecutive iterations, replicating the same time frame as a volunteer scan. As in the previous case, the system does not move.  Figure 5 shows the 200 corresponding sensitivity curves, which sit mostly below −80 dB except for the extremes of the frequency band. The corresponding phase variation is below 0.5° with an increase towards the end of the spectrum. The correlation between the phase variation and the sensitivity is obvious from Figure 5. Overall we observe that, due to the drift inherent in the VNA, a 30-minute time span between measurements decreases the microwave measurement sensitivity by roughly 10 dB. 

### 3.3. Microwave Measurement Perturbation Immunity

A perturbation is defined as any phenomena (internal or external) that will induce unpredictable interference in the measured signals and thus affect the measurement sensitivity. A number of perturbation sources are identified and the solutions to mitigate their effect are described.

**Figure 6:**
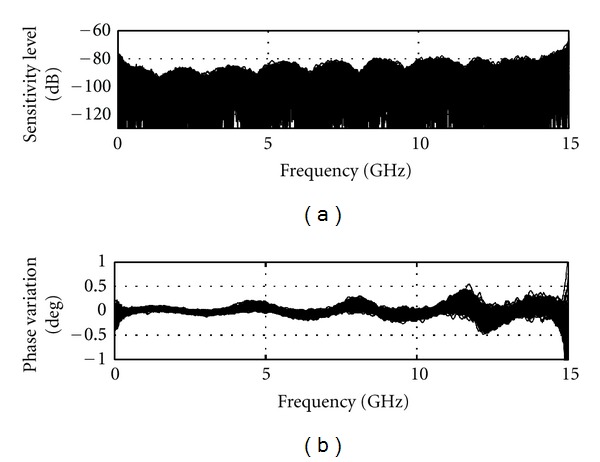
Sensitivity calculated based on a pair of full TSAR scans (200 positions) with a broadband load instead of the antenna. The load is physically moved with the prototype system to 200 locations.

The first perturbation arises from the change of the cable response. As the antenna is moved to various locations, the cable shape is changed which predominantly affects its phase response. To reduce the negative effect on the sensitivity, a guiding system shown in Figure 1 has been implemented. This system helps to ensure that the cable position is repeatable when the antenna is positioned and repositioned at a certain location. Identical cable positions translate to similar electrical responses that can be removed during the calibration process. The performance of this technique is illustrated in Figure 6, which shows the sensitivity calculated when the system is moved through two full TSAR scans (200 positions) with the broadband load attached instead of the antenna. When comparing with the corresponding static sensitivity (Figure 5), we observe only a slight increase of the phase variation, which translates to a fairly limited degradation of the sensitivity. An additional set of results is generated without any cable compensation by taking the difference between the 200 measurements and one selected measurement from the second scan. In this way, the cable position is different for each of the measurements in a given pair. For this scenario, the sensitivity sits at around −70 dB, so we estimate that the cable guiding system improves the sensitivity by about 10 dB. 

**Figure 7:**
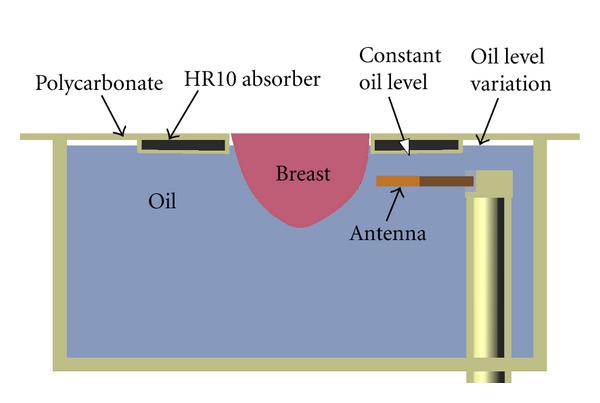
Profile view of the specially designed lid.

The other perturbations are related to the signals detected by the antenna. The reflections from the breast are of interest, while reflections from other objects or sources can be subtracted during the calibration process as long as they are stable between measurements. However, any unpredictable signals that cannot be removed with the calibration process are considered as perturbations and need to be minimized. The unwanted signal sources have been classified into three groups: (a) lab environment reflections (room, equipment, people, etc.), (b) immersion liquid movement, and (c) general electromagnetic smog. Different mechanisms are implemented to alleviate these perturbations. First, the lab environment reflection (a) is easily removed using the time gating implemented in the VNA. The measured data are gated between 0 and 3.6 ns in order to remove reflections that originate from outside of the measurement tank. The immersion liquid movement (b) is induced by the movement of the tank itself but most predominantly by the fluctuation of the tank volume due to the moving arm displacement. As the volume changes, the liquid level changes and creates reflections that cannot be replicated since these reflections are also affected by the volume of the breast itself. To minimize this effect, the tank lid is designed with additional material added around the hole through which the breast extends (Figure 7). This keeps the liquid level constant in the vicinity of the antenna aperture, while allowing fluctuation in liquid level behind the antenna where radiation is an order of magnitude less. This additional region consists of a polycarbonate shell filled with HR10 absorber (Emerson and Cuming Microwave Products, Randolph, MA, USA). 

Finally, the electromagnetic smog (c) is generated by electrical apparatus around the lab and the outside world. To increase the electromagnetic immunity, absorbers are placed at strategic locations around the measurement tank in conjunction with shielding material. 

**Figure 8:**
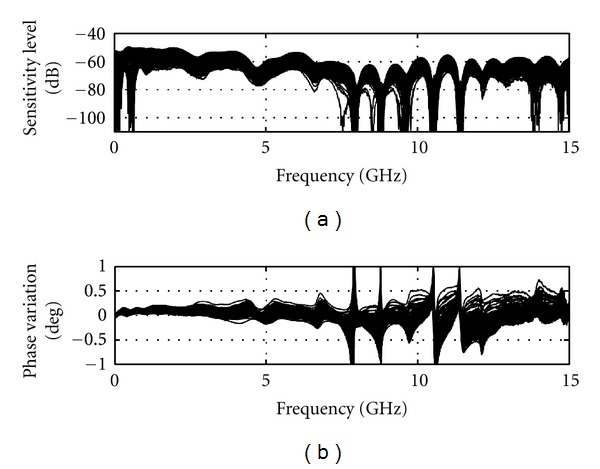
Sensitivity calculated based on a pair full TSAR scan (200 positions) with the antenna attached and immersion medium present (as per a volunteer scan).

**Figure 9:**
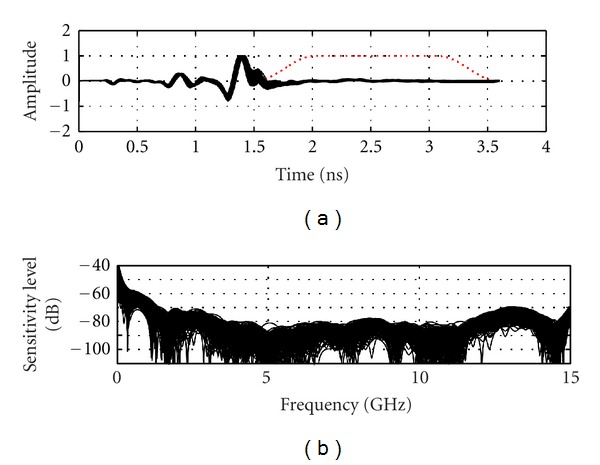
Time domain representation and noise level of the later-time antenna response (under dashed window).

Figure 8 shows the typical sensitivity of the TSAR prototype, when the previously mentioned techniques are in place, the antenna is attached and the immersion liquid is present. When compared to Figure 6, a significant decrease in magnitude sensitivity is noted, resulting in sensitivity between −50 and −60 dB, while phase variation increases slightly. The very large peaks in the phase variation happen at resonances where the phase changes drastically while being difficult to resolve by the VNA due to the weakness of the reflected signal. Overall, a sensitivity reduction of 30 dB is observed. As the reflection coefficients of the broadband matched load and antenna are around −30 and −10 dB, respectively, the phase variation intrinsically has greater impact on the sensitivity with the antenna attached. However, since the BAVA-D ringing is extremely small, the increase in reflection is mostly located in the antenna structure, as shown by the time domain representation in Figure 9. The antenna structure ends at approximately 1.5 ns in time and only the components of the signal beyond this point are significant for imaging purposes. We use a Tukey window, shown in Figure 9, to evaluate the sensitivity of the signal occurring after the antenna structure. As shown in Figure 9, the sensitivity sits overall between −70 to −80 dB. The lower frequencies are ignored since the antenna does not radiate well below 2 GHz. Based on these values, we assess that 10 dB are lost in sensitivity when the antenna is attached instead of the load (i.e., compared to Figure 6). 

Overall, the TSAR prototype may be expected to have reflection sensitivity between −70 to −80 dB. The VNA itself demonstrates a sensitivity level of −90 dB and is therefore more than capable of measuring signals greater than the reflection sensitivity. Moreover, numerous technical challenges arise when consistent performance needs to be maintained while scanning around a cylindrical volume. The TSAR system has demonstrated excellent mechanical accuracy and repeatability, and the modifications to the prototype system aimed at ensuring measurement sensitivity appear to enhance performance. This has resulted in a prototype system that demonstrates acceptable performance for our application. 

## 4. Hemispherical Breast Model 

The basic performance of the prototype system has been examined, however it is also of interest to validate reflections from test objects by comparing simulated and measured results. First, the hemispherical breast model used for this investigation is described. Reflection data are analyzed in relation to the previously presented performance metrics. Images created with simulated and measured data are also discussed. 

**Figure 10:**
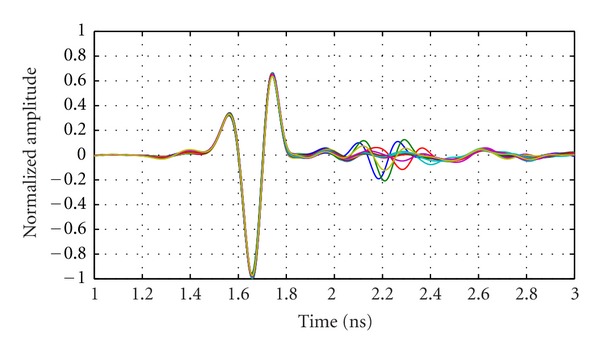
Measured reflections from the breast phantom at 20 antenna locations encircling the model. Measurements are collected with the antennas positioned at the same *z*-location as the inclusion.

The model used for this work has a relatively simple shape and composition and is described in detail in [18]. The model consists of a cylindrical section (diameter of 10 cm) attached to a hemispherical section with radius of 5 cm. A series of rings is located on the hemisphere in attempt to mimic the shape of the nipple. The model is made of a low-loss dielectric material with relative permittivity of 15. This phantom contains a cylindrical inclusion consisting of a Teflon rod of 7.9 mm diameter and 19.4 mm length. The inclusion is located in the hemispherical region at a radial distance of 25 mm from the centre of the model. The model is placed in the scanner, and the BAVA-D antenna is used to obtain measurements. For a full scan of the model, the antenna is scanned to 7 rows (vertical locations) separated by 1 cm and to 20 locations per row. A second scan of the empty tank is performed for calibration purposes. The antenna locations are the same as those used in the scan of the phantom. The reflections recorded with an empty tank are subtracted from those recorded with the model present. Reflections collected at the row of antennas located at the center of the inclusion are shown in Figure 10. Dominant reflections are expected from the oil/phantom interface and are shown to be very similar for one row of measurements. The response from the inclusion is also evident after 2 ns for antennas located closer to this object. 

**Figure 11:**
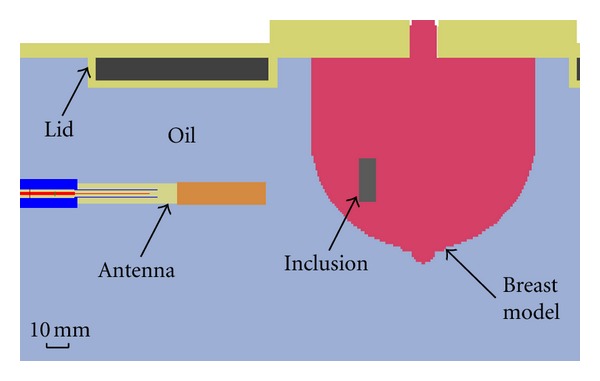
Cross-section through the voxelized simulation model of phantom showing components of the prototype included to more accurately model reflections.

**Figure 12:**
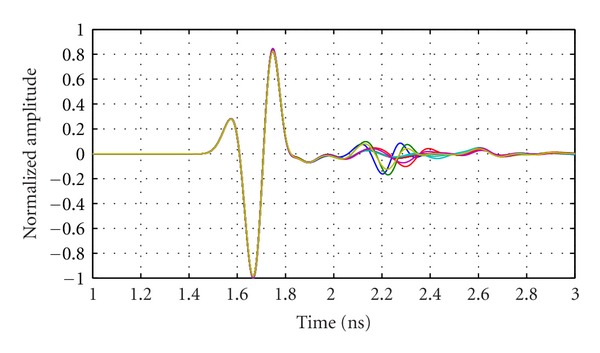
Simulated calibrated reflections from the breast phantom at 20 antenna locations encircling the phantom. Reflections are simulated with the antennas positioned at the same *z*-location as the inclusion.

Next, simulations are performed in order to gain further insight into the measured data. The detailed simulation model includes aspects of the system that are expected to influence the reflected signals. Specifically, the model includes a replica of the breast phantom, a BAVA-D antenna, the top of the tank, and the immersion liquid (Figure 11). Simulations are performed using SEMCAD (SPEAG, Zurich, Switzerland), which uses a finite difference time domain (FDTD) solver, and the antenna is excited with the UWB pulse described in (1). Results obtained with the breast phantom are shown in Figure 12 for one row of antennas (also located at the level of the inclusion in the phantom). Similar to Figure 10, dominant reflections are expected from the oil/phantom interface and are shown to be very similar for one row of simulations.

To investigate the similarity between the dominant reflections with measured and simulated data, we apply Tukey windows to isolate the first reflection (mean extent of 0.83 ns and positioned relative to the maximum absolute response in each signal). Correlations between these windowed reflections for the data shown in Figures 10 and 12 are on average 0.99. For the 140 antenna locations used to scan the phantom, simulations and measurements show mean correlation of 0.98 with standard deviation of 0.014. 

**Figure 13:**
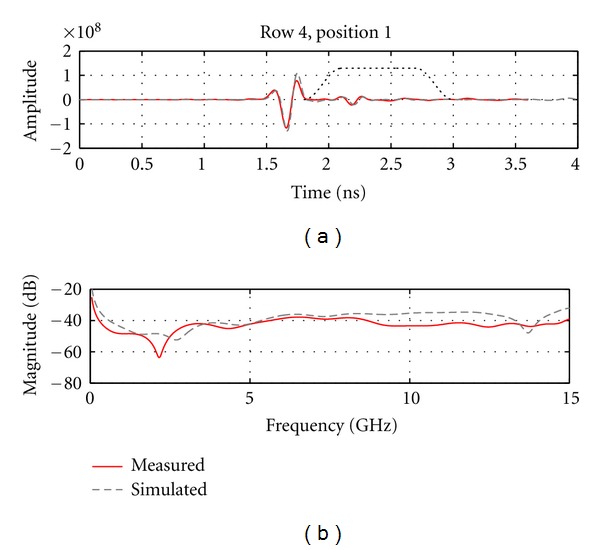
(a) Calibrated reflection from the breast model recorded by the antenna situated the closest to the inclusion. Dotted line shows the extent of the Tukey window that is used to isolate the later-time response. (b) The frequency response of the later-time component of the signals.

**Figure 14:**
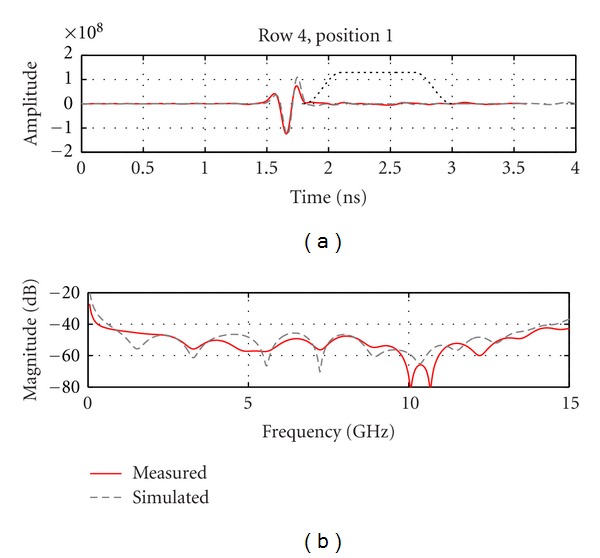
(a) Calibrated reflection from the breast model recorded at the same position as in Figure 13 but without any inclusion present. Dotted line shows the extent of the Tukey window that is used to isolate the later-time response. (b) The frequency response of the later-time component of the signals.

Next, we examine and compare the later-time responses from simulation and measurement models by again using a Tukey window to isolate reflections occurring after the dominant reflection.  Figure 13 shows results for an antenna located the closest to the inclusion, while Figure 14 shows results for an antenna at the same location but without any inclusion present in the breast model. We note that the simulated and measured data are in good agreement for the case containing the inclusion, as both time and frequency domain results are similar. When the inclusion is present, a reflection of about −40 dB is reached, which is easily detected given the sensitivity of our measurement system. Without an inclusion present (Figure 14), a lower reflection is noted in the later-time response. On average, the reflected signal without an inclusion present is 7 dB lower for the measured data and 11 dB lower for the simulated data. The signals magnitudes in Figure 14(b) are very similar between simulations and measurements while still within the sensitivity of the system. This suggests that part of these smaller responses are indeed components of the reflected signals, likely originating from subtle sources such as the late-time response from the interface between oil and the model. Therefore, the TSAR prototype system demonstrates the ability to accurately record these fine details caused by larger reflected signals. 

**Figure 15:**
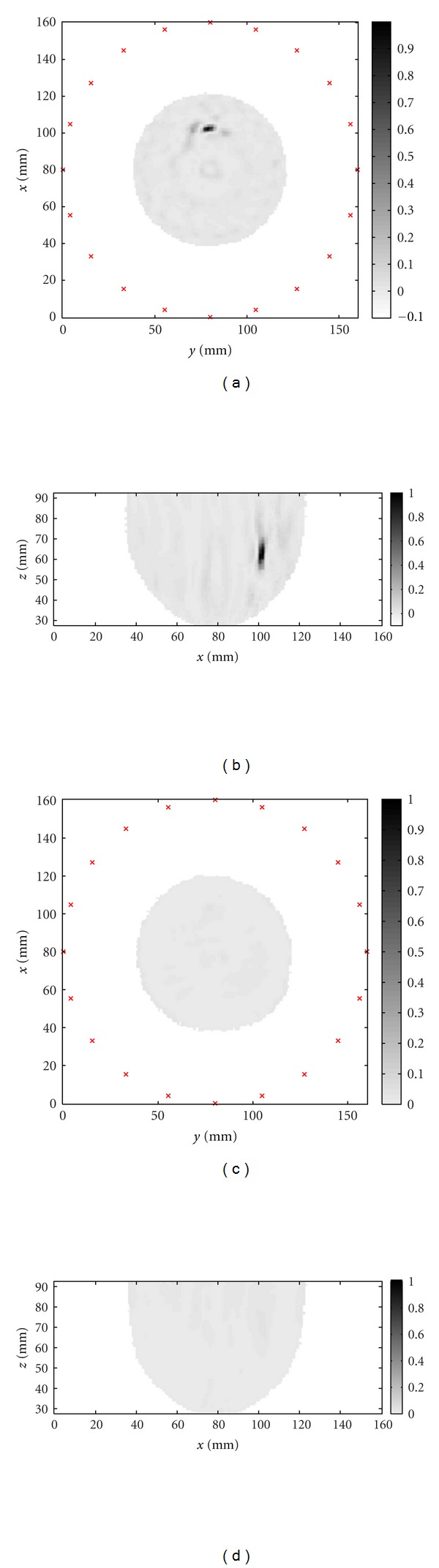
Images of the hemispherical model created from measured data: (a) slice through the inclusion location perpendicular to the axis of the cylinder and (b) slice through the inclusion location parallel to the axis of the cylinder. The images for the phantom without the inclusion are shown in (c) and (d).

Finally, the simulated and measured data are used to create images. Phantoms with and without the inclusion are imaged and results obtained for measured data are shown in Figure 15. Similar results are obtained for simulated data, however images are not shown as the results appear very similar to those in Figure 15. The inclusion is easily detected and localized, and maximum response of the inclusion is located 23 mm from the center of the model. The location error likely results from challenges in orienting reflections precisely in time, as well as the discrete nature of the imaging procedure. The maximum response of the inclusion is compared to the response at the same location in the inclusion-free image. For measured data, the response with the inclusion is 14.1 dB greater than the inclusion-free case, demonstrating the enhancement of the inclusion response achieved through both reduction of common reflections and coherent summation via the focusing algorithm. For simulated data, the ratio is 47.4 dB, demonstrating the higher similarity between the simulated reflections, as well as the inherent differences between measurements and simulations. 

Overall, the investigation of the breast model indicates good agreement between simulations and measurements, which validates the accuracy of our measurements. The response from the inclusion is easily measured given the sensitivity of our system, and images clearly detect and localize the inclusion. 

## 5. Initial Measurements with a Volunteer

The work with the hemispherical model provides an assessment of the similarities between simulated and measured data for the TSAR prototype. These results suggest that simulations of a realistic breast model may provide a means to interpret measured reflections from human volunteers, as the dominant reflections are expected to be similar for measurements and simulations. 

A detailed analysis of a volunteer study is performed, using TSAR and MR scans. A volunteer is scanned with the TSAR prototype using the scan pattern presented in Table 2 (note that the origin of the vertical axis is coincident with the bottom of the lid) and measurement parameters discussed in Section 2. MR images are collected with a 1.5 Tesla Siemens Sonata MR Scanner and breast coil. The scanning sequence is T1-weighted (Gradient Echo VIBE with variant SP/OSP). With this sequence, fat is suppressed and glandular tissue has higher pixel intensity in images. The pixel size is 0.4297 mm × 0.4297 mm × 1.2 mm, and 112 images are collected for this volunteer. 

**Figure 16:**
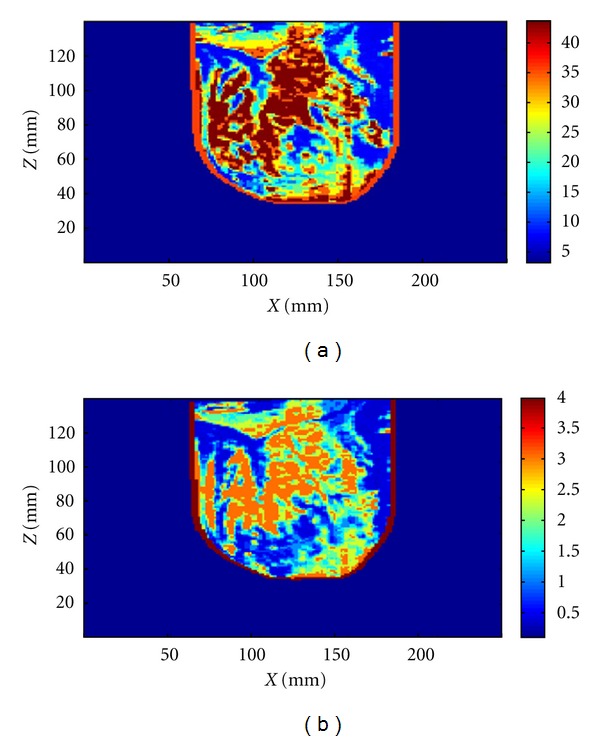
Permittivity (a) and conductivity (b) distribution in cross-section of a simulated breast model after adjustment for floatation in oil.

To permit us to compare simulated and measured data, the MR images are translated into a model suitable for use with SEMAD. Mapping pixel intensity in MR images to electromagnetic property values involves several approximations, and the procedure used to create the breast model follows that described in [10] with the breast interior represented with 16 tissues. A cross-section of the realistic model used in simulations is shown in Figure 16. The MR and TSAR scans are both collected with the volunteer in the prone position, however the extent and shape of the breast differ when comparing the two systems. The key difference is that breast also floats in the oil used as the immersion liquid in the TSAR scanner. To compensate for this effect, the voxel size in the *z*-direction (Figure 16) is reduced from 0.4297 to 0.36 mm. To approximate the locations at which the measurements are collected, the nipple is used as a landmark and we assume that, at the antenna row closest to the top of the tank, the breast is centered in the scanner. Specifically, the location of the row of antennas closest to the nipple is determined from digital images collected during the TSAR scan. This information is used to position the antennas in simulation, and the scan pattern described in Table 2 is replicated. Reflections from the breast model are simulated using the pulse in (1). 

**Figure 17:**
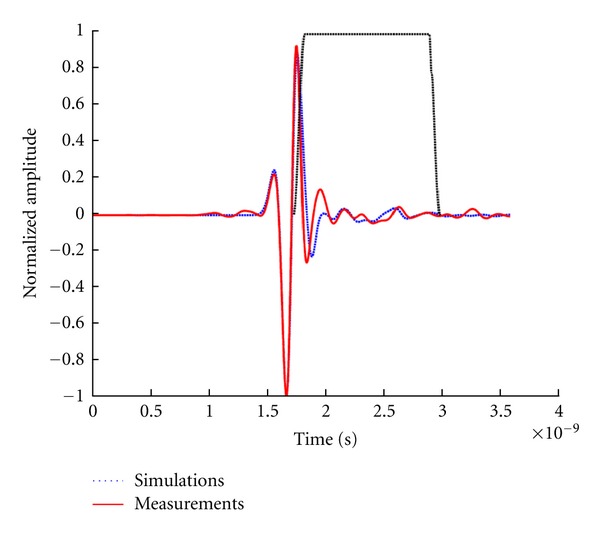
Reflections recorded in experiments and simulations. A Tukey window applied to isolate late-time reflections is shown. The reflections are normalized to the maximum value and shifted in order to align the skin responses.

The measured data from the volunteer are compared with simulations of the volunteer-specific model.  Figure 17 shows normalized reflections from a simulation of the compressed breast and the corresponding experimental measurement.  Figure 17 shows that the signals are in reasonable agreement with differences likely resulting from the fact that the simulated skin is modeled as a 2.14 mm layer, while the thickness of the skin approximated from the MR images ranges between 1.5 mm and 3.0 mm. Similar results are observed for the majority of antenna locations, as confirmed by calculating the correlation between the measured and simulated signals. For 116 out of 120 signals, the correlation is 0.9 or better, demonstrating the similarity between measured and simulated skin reflections recorded as the antenna is scanned around the breast. The outliers likely originate from areas of the model where skin thicknesses are significantly different when compared to the actual skin thickness of the volunteer. Therefore, the TSAR prototype is capable of measuring reflections from volunteers and comparison of measurements and simulations suggests that the measured reflections are reasonable. However, detailed analysis of later-time reflections is not considered, as numerous differences between the model and volunteer are present (e.g., breast shape differs from MR to TSAR and antenna locations are approximated). This makes this comparison of small later-time reflections extremely challenging. 

## 6. Conclusions

In this paper, a prototype system for monostatic radar-based imaging of the breast is described. This system scans a single UWB antenna around the breast in order to collect data, therefore differing from prototype systems for multistatic radar-based imaging and tomography. The paper first focuses on evaluating the performance of the system, as this is key for gaining insight into the capabilities and limitations of the prototype. For example, the motion of the sensor impacts the system performance, so the accuracy and repeatability of sensor positioning are assessed, showing minimal errors. Microwave measurement sensitivity is defined as the differences between two reflection measurements and is used to examine the effects of time-delay between measurements, system motion and cable flex. Differences in measurements with a broadband load attached show that time delay and motion do degrade the sensitivity. By controlling cable positioning, improving measurement environment repeatability and applying techniques such as time-gating the reflections, the microwave measurement sensitivity during the TSAR scan is assessed to be between −70 and −80 dB. In addition, the metrics examined appear to be informative and may be used to evaluate performance of monostatic radar-based imaging systems. 

Once the system performance is evaluated, simulations and measurements of a simple phantom are compared. Although much work with both simulations and measurements has been reported for microwave imaging systems, there are only a few reports directly comparing these results. Both early and late-time reflections recorded from a simple phantom show very good agreement. Moreover, reflections from homogeneous phantoms are compared with reflections from phantoms containing inclusions, demonstrating that the response of the inclusion is easily detected given the sensitivity of the system. In addition, the measurement of the weaker later-time reflections from the phantom correlate with simulated results, bringing confidence to the measurement accuracy. The resulting images indicate the inclusion is easily detected and localized. 

Finally, a scan of a volunteer is described and analysed. In order to interpret the reflections, a volunteer-specific breast model is created. The early-time reflections in simulations and measurements are in excellent agreement, given the known differences between the volunteer and model. This provides confidence that the measured signals correspond to reflections from the breast tissues. 

Measurement perturbation due to breast movement induced by volunteer movement or by potential turbulence during sensor displacement were not considered in this paper. Given the length of the scan time (30 minutes), patient movement is expected. However, considering the resolution of a biomedical microwave imaging system (subcentimeter scale), small movements are not expected to significantly affect image quality. For comparison, breast MRI can take up to 40 minutes while achieving image resolution in the millimeter scale. It is also important to observe that for both modalities the patients lie in a prone position with the chest wall resting on the breast coil or the measurement tank lid. In this configuration, movement occurring during patient breathing has only a limited impact on the breast position as the breasts do not significantly move relative to the chest wall. Based on the volunteers scanned so far (12), no significant breast movements have been observed between the digital images recorded at each antenna position. Breast movement during antenna displacement or while the VNA is sweeping could not be assessed visually. However, the good correlation between the measured signals and simulated counterpart using the patient specific model suggests that movement during the VNA sweep is minimal. 

Future work includes improving the agreement between the simulated and measured reflections from volunteers and patients, especially the later-time responses. For example, the laser surface measurement of the breast may be used to more accurately deform the MR-based breast model. Combined with knowledge of microwave measurement sensitivity, simulations of the realistic breast models may be used to gain insight into the ability to detect a range of tumors located at different locations in breasts containing a variety of tissue distributions. 

## Figures and Tables

**Table 1:** Measured mechanical play for the elevation axis. All values are in mm.

Test Iteration	Without software compensation		With software compensation	
	Downward	Upward	Downward	Upward
1	**0.11**	0.07	0.02	0.01
2	0.10	0.10	−0.01	−0.01
3	0.10	0.08	−0.03	−0.01
4	0.06	0.07	−0.02	−0.01

**Table 2:** TSAR scan parameters.

Parameter	Value
Vertical scan extent (mm)	−20 to −70
Number of rows	6
Separation between rows (mm)	10
Number of antennas per row	20
Separation of neighboring antennas (°)	18
Rotational offset between rows (°)	6
